# Drug Repurposing at the Interface of Melanoma Immunotherapy and Autoimmune Disease

**DOI:** 10.3390/pharmaceutics15010083

**Published:** 2022-12-27

**Authors:** Alf Spitschak, Shailendra Gupta, Krishna P. Singh, Stella Logotheti, Brigitte M. Pützer

**Affiliations:** 1 Institute of Experimental Gene Therapy and Cancer Research, Rostock University Medical Center, 18057 Rostock, Germany; 2 Department of Systems Biology and Bioinformatics, University of Rostock, 18057 Rostock, Germany; 3 Department Life, Light & Matter, University of Rostock, 18059 Rostock, Germany

**Keywords:** melanoma, metastasis, autoimmune disease, cancer immunotherapy, tumor immune microenvironment drug repurposing, irAE, E2F1, structure-based pharmacophore modeling

## Abstract

Cancer cells have a remarkable ability to evade recognition and destruction by the immune system. At the same time, cancer has been associated with chronic inflammation, while certain autoimmune diseases predispose to the development of neoplasia. Although cancer immunotherapy has revolutionized antitumor treatment, immune-related toxicities and adverse events detract from the clinical utility of even the most advanced drugs, especially in patients with both, metastatic cancer and pre-existing autoimmune diseases. Here, the combination of multi-omics, data-driven computational approaches with the application of network concepts enables in-depth analyses of the dynamic links between cancer, autoimmune diseases, and drugs. In this review, we focus on molecular and epigenetic metastasis-related processes within cancer cells and the immune microenvironment. With melanoma as a model, we uncover vulnerabilities for drug development to control cancer progression and immune responses. Thereby, drug repurposing allows taking advantage of existing safety profiles and established pharmacokinetic properties of approved agents. These procedures promise faster access and optimal management for cancer treatment. Together, these approaches provide new disease-based and data-driven opportunities for the prediction and application of targeted and clinically used drugs at the interface of immune-mediated diseases and cancer towards next-generation immunotherapies.

## 1. Introduction

Metastasis is the leading cause of cancer-related deaths worldwide and depends on molecular alterations in the cancer cells themselves, but also on the tumor immune microenvironment (TIME). Targeted single-agent therapies for patients with molecularly defined tumors have transformed cancer treatment. However, effective treatments still do not exist for many patients, especially with metastatic cancers, and pre-existing or acquired resistance limits the clinical utility of even most advanced drugs. Cancer therapeutics can directly exploit vulnerabilities in tumor cells and beyond, alter components of the tumor microenvironment (TME). In search for the next-generation therapies, the best possible combination of target-oriented anticancer drugs and immunotherapy are likely the most effective treatment modality. Immune checkpoint inhibitors have demonstrated clinical activity, with efficacy and significant survival benefit for anti-programmed cell death protein 1 (PD-1), anti-cytotoxic T-lymphocyte antigen-4 (CTLA-4), or combined immunotherapies in patients with metastatic melanoma and other tumor entities. However, despite recent breakthroughs, new therapeutic strategies are urgently needed, especially for melanoma patients with pre-existing autoimmune diseases (AD) or patients developing autoimmunity upon immunotherapy. The major challenge is to efficiently identify potential drug candidates or combinations thereof based on comprehensive molecular analyses, and to maximize the likelihood that drugs discovered by biochemical or phenotypic methods will lead to clinical efficacy and improved disease management. Consequently, both researchers and clinicians are interested in an accelerated process when searching for cancer therapeutics. One way is to identify new therapeutic uses for existing drugs, a strategy generally referred to as drug repurposing or repositioning. Drugs and drug candidates for novel therapeutic indications act on new targets outside the original scope also called off-target profiles. The main purpose of drug repositioning is to take advantage of the safety profiles and pharmacokinetic properties of well-studied agents approved in clinical trials. Further, repurposing promises faster access to active ingredients at lower costs. Currently, drug development from scratch can take up to 15 years and the costs to approve and launch a new drug starting from hit selection in vitro, are estimated USD 2 to 3 billion, and these amounts continue to skyrocket [1].

## 2. Drivers of Metastatic Melanoma as Hallmarks of Cancer Progression

Melanoma is the most aggressive form of skin cancer. Characteristics for advanced melanoma cells with metastatic potential are molecular alterations in gene transcription programs and protein functions that occur to a large extent through epigenetic reprogramming and changes in the TME. The term ‘metastatic potential’ encompasses any combination of cancer phenotypes that enable metastatic dissemination including motility, immune evasion, and the ability to survive in the bloodstream and proliferate at distant sites [2].

### 2.1. E2F1 as Driver and Therapeutic Target of Melanoma Metastasis

In search of relevant vulnerabilities underlying melanoma metastasis in conjunction with the TME, the cellular transcription factor E2F1 was identified as a key inducer of cancer cell dissemination. We and others demonstrated that progression of cancers such as melanoma [3,4], breast [5], and bladder cancer [6,7] is catalyzed by the abundant expression of E2F1 and E2F1-mediated activation of downstream pro-metastatic gene regulatory networks (GRNs). Here, epigenetic reprogramming occurs considerably more often. E2F1 belongs to the E2F transcription factor (TF) family and plays a physiological role in controlling the cell cycle. In tumors, however, E2F1 exhibits a janus behavior across the successive stages of carcinogenesis. Early in tumor development this TF activates tumor-suppressive pathways by initiating apoptosis. In advanced stages, E2F1 switches duties and is rewired to networks that promote metastatic spread. This includes resistance to therapy [8,9], angiogenesis [10], extravasation [11], EMT [7,12], metabolic reprogramming [13], and genomic instability [14]. E2F1, therefore, can be considered as a potential new gold standard for melanoma metastasis.

Tumor type-specific gene signatures were detected, showing that highly expressed E2F1 in combination with TGFBR1, and FGFR1 is causatively implicated in EMT-driven invasiveness [7,15]. In addition, several protein coding genes (PCGs), miRNA, and lncRNA genes have been identified as constituents of E2F1-activated pro-metastatic GRNs [7,13,14,16]. Within the E2F1-governed GRNs, non-linear feedback and feedforward regulatory motifs are formed among various regulatory network layers entailing these protein-coding and non-coding RNA genes [7,12,13,14]. Such regulatory motifs which are commonly encountered in cancer networks [7] induce a whole range of dynamic behaviors. Mechanistically, the aggressive activity of E2F1 largely depends on the spatiotemporal availability of transcriptional coregulators that enhance its transcription programs by forming protein–protein interaction (PPI) complexes to favor expression of genes that promote a metastasis-prone TME. In line with this, it was recently shown that coactivators overexpressed in highly aggressive cancer stem cells (CSC phenotype) can direct E2F1 to enhance the transcription of metastasis-related genes [9,10,11,13,14]. Here both, the epidermal-growth factor receptor (EGFR) and vascular-endothelial-growth factor receptor 3 (VEGFR-3) genes, are directly transactivated by E2F1 and act as transcriptional coregulators in a feedback circuit on target genes to enhance invasion and angiogenesis [3,10]. Thus, targeting this PPI might be reasonable to complement standard anti-angiogenic treatment of cancers with deregulated E2F1 and can play a role in drug repurposing (see Section 7).

### 2.2. Role of the TME for Metastasis

The complex and dynamic interplay between intratumoral heterogeneity and TME massively influences the treatability of metastasis-prone cancer cells. Interactions are established among heterogeneous cancer cell subpopulations and cellular and non-cellular components of the TME, forming complex pro-metastatic networks. Many signals from the TME, such as chronic inflammation and secretion of inflammatory cytokines, hypoxia, and perivascular niches that regulate the capacity of proliferation and differentiation, are necessary for the maintenance of CSCs, self-renewal, angiogenesis, and cancer spreading [17,18]. Additionally, given that stromal cells within the TME, compared to the heterogeneity in cancer cells, are genetically stable and are thus likely to be less susceptible to classical mechanisms of therapeutic resistance, microenvironment constituents can be key regulators of the sensitivity to CSC-targeted therapies [19].

Moreover, production of neuronal signaling molecules by cancer cells modifies the dynamics of cellular interactions within the TME including immune, endothelial, and neuronal cells expressing surface receptors responsive to them [20,21]. Since neurotrophins and neurotransmitters are recognized by cancer, neuronal, and immune cells, they could act as common signaling molecules for complex cell–cell interactions among each other. Beta-adrenergic signaling, for instance, affects secretion of the pro-inflammatory cytokines IL-1, IL-6, and IL-8, VEGF, and matrix metalloproteinases, facilitating angiogenesis and tissue invasion, whereas acetylcholine stimulates secretion of the anti-inflammatory, immunosuppressive cytokines IL-10 and TGF-beta, and inhibits the production of pro-inflammatory IL-1b, TNF-α, and IL-12 cytokines [22]. In addition, high-neuroendocrine subtypes of small cell lung cancer show decreased immune cell infiltration defined as ‘immune desert’ phenotype, while low-endocrine subtypes present increased immunogenicity, that are contrarily termed ‘immune oasis’ phenotype, and more likely to respond to immunotherapies [23]. These and other data suggest that sustained release of neuronal signaling mediators in the tumor milieu promotes metastasis by modulating the immune system. Therefore, particular attention in terms of therapeutic management should be also focused on the cancer–neuronal crosstalk and its impact on the TIME to prevent metastasis and improve immunotherapy [15,24,25,26,27].

Towards precision immuno-oncology, a suite of experimental, multi-omics, and theoretical methods have been developed to assess cancer specific biological pathways and their interaction with the TME and to unveil how their components are rewired towards metastatic fates, ultimately leading to a revolution of the biomarker landscape and skyrocketing new drug targets. In this context, it has been revealed that key hallmarks of malignancies, such as metastasis and immunity, are not controlled by a single signaling pathway. Concomitantly, the TME consists of multiple specialized microenvironments that overlap and constantly interact with each other. Consequently, conventional drugs with new applications of targeting multiple cancer traits or combination therapies are likely advantageous for avoiding progression of resistance and short-term use in clinical practice [28]. Such approaches could catalyze a paradigm shift in personalized cancer patient treatment and drug repurposing.

## 3. Immunotherapeutic Strategies against Metastatic Melanoma

Melanoma as a cancer with poor prognosis has the ability to evade recognition and destruction by the patient’s immune system [29], and both, the intrinsic biology of the tumor and its immune microenvironment are critical for evading the host’s immune surveillance. This tumor cell property is determined by the relative proportions and absolute amounts of different immune cell types within the TME and includes T cells as well as B cells, dendritic cells (DC) and natural killer cells (NKs), tumor-associated macrophages, myeloid-derived suppressor cells (MDSCs), tumor-associated neutrophils, granulocytes, mast cells, cancer-associated fibroblasts (CAFs), adipocytes, vascular endothelial cells, and pericytes [30]. The dominant presence of regulatory T cells (Tregs), for example, results in an immune tolerant phenotype. Moreover, a complex interplay of immune cell types, expressed antigens, and secreted factors alters the ability of the immune system to eradicate tumor cells. A detailed description of immune evasion mechanisms is outlined in the recent review by Kim and Cho [31].

Ultimately, immunotherapeutics stimulate an individual’s immune system to identify and destroy the cancer cells and to circumvent inadequate immune responses. Here, they ally with the immune cells to shrink the primary tumor and to establish durable responses against circulating tumor cells that might lurk beyond the site of tumor onset. The cancer immunotherapy toolbox contains four operating principles. Amongst the four, the checkpoint inhibitors act by releasing the brakes that prevent T cells from attacking and killing cancer cells. On the humoral side, monoclonal antibodies directed against specific antigens are made to bind to cancer cell surface antigens. So-called cancer vaccines were designed to boost the immune system’s inherent responses to the tumor. The other principle includes the cell-based therapy approaches for instance chimeric antigen receptor (CAR) T-cell therapy. The latter, a process termed adoptive transfer, acts in a way that T cells are taken from a patient’s tumor, expanded ex vivo, and/or genetically engineered and re-administered [32].

### 3.1. Immune Checkpoint Inhibitors and Other Immunotherapeutic Approaches

The entity of successful immunotherapy application includes, but is not limited to, melanoma, especially if diagnosed at late stages [33]. To treat metastatic melanoma the immunomodulatory cytokine interleukin-2 (IL-2) was approved as early as 1998 [34]. After 2011, the FDA approved the checkpoint inhibitors pembrolizumab, nivolumab, and ipilimumab to treat of metastatic melanoma [33]. Clinical outcomes improved significantly, in conjunction with these inhibitors either as mono- or in combination therapy regimens [35]. Immune checkpoint inhibitors (ICI) have shown clinical activity in advanced melanoma with significant survival benefits and response rates of 19% for the anti-CTLA-4 antibody ipilimumab and 36–44% for the anti-PD-1 antibodies nivolumab and pembrolizumab [36]. Combined CTLA-4 and PD-1 blockade has achieved an unprecedented 5-year overall survival of over 50% [37].

Aiming at immunomodulation of the TME, much attention has also been paid to cell-based strategies. CD4^+^ FoxP3^+^ Tregs, which exert a suppressive effect on effector T cells implicated in tumor surveillance, have been shown to account for a large proportion of tumor-infiltrating lymphocytes (TILs) in melanoma and breast cancer that do not respond to ICI [38]. Thus, the attenuation or abrogation of tumor-infiltrating Treg function, although occasionally associated with increased immune-related adverse events (irAEs) [39], has been a subject of great interest in the context of immuno-oncology therapeutics [40]. In addition, biological and small molecule-driven alteration of Tregs is under intense study [41]. Wang et al. (2018) found that their function in tumor infiltrates is dependent on the Enhancer of Zeste Homolog 2 (EZH2) protein [42]. Pharmacological inhibition of EZH2 resulted in Tregs acquiring a pro-inflammatory activity that enhanced immunity to cancer. Another tumor intrinsic axis of major pharmacological interest for cancer therapy is the vascular endothelial growth factor (VEGF)/VEGF receptor (VEGFR) system. In particular, blockade of this pro-angiogenic pathway through VEGFR2 kinase inhibition has been shown to be effective in numerous cancers [43], reducing immunosuppression by preventing recruitment of immature DC, myeloid-derived suppressor cells, and Tregs [44,45]. Anti-angiogenic agents targeting this axis promote a pro-inflammatory microenvironment [46].

### 3.2. Shortfalls of Immunotherapies—irAEs and Autoimmune Disease

In recent years, various cancer immunotherapies showed a remarkable success. However, only up to 20% to 30% of cancer patients have benefited from these treatments, while the rest were either partial or non-responders. Especially, the latter is related to factors such as CD8^+^ T cell density in the TME, monocyte frequency, tumor heterogeneity, neoantigen load, and the composition of patient’s gut microbiota [35]. Further challenges of immunotherapy include immune-related toxicities or irAEs, an activation of the immune system and inflammatory response against healthy tissue. In particular, 85% of melanoma patients under ipilimumab treatment have suffered from irAEs, with more than one-third discontinuing therapy or requiring additional systemic treatment to manage side effects [47].

Moreover, immunotherapy faces limitations in patients with both an overactive (AD patients), or a suppressed (organ transplant recipients) immune system. Cancer patients with pre-existing autoimmune diseases who receive ipilimumab treatment frequently experience disease flares and exacerbations, that, such as patients with irAEs, either require additional immunosuppression or therapy discontinuation [48,49]. Eventually, the ability to predict the risk of severe irAEs could provide a deeper understanding of the underlying mechanisms and predisposing factors, allowing physicians to improve and personalize cancer immunotherapy for the patient. While modern immunotherapeutics have dramatically improved survival and quality of life for many patients, not all cancers are equal, and currently, very few predictors of response and toxicity do exist. At the present level, it is becoming more difficult to enhance the efficacy of clinically established combination therapies. Considering treatment-related toxicity and the fact that irAEs can be associated with mortality and significant lifelong morbidity, predictors and novel strategies that are not only safer for such patients but are also able to manage cancer along with a co-existing immune-related disease, are urgently needed.

## 4. A Look at the Interface of Cancer and AD

Frequently, cancer patients do experience disorders including inflammation, but also autoimmune diseases either occurring as predisposing, as pre-existing, or as intercurrent conditions [50,51,52]. Intriguingly, these diseases seem to be etiologically interrelated with one another. On one hand, cancer has been correlated to chronic inflammations. Further, interconnections between cancer and autoimmune diseases exist, as some autoimmune disorders predispose to neoplasia [53,54]. Patients suffering from AD for example dermatomyositis, inflammatory bowel disease (IBD), rheumatoid arthritis (RA), psoriasis, systemic lupus erythematosus (SLE), or Sjögren syndrome may have increased risks for malignancies with a dependency between an AD and certain types of cancer [55]. Although generally, AD do not necessarily initiate neoplastic changes, they are phenotypic manifestations of a deregulated if not a dysfunctional immune system, and thereby prone for cancer development [56]. Cancer immunotherapy triggers, vice versa, autoimmunity towards one or another tissue and can lead to conditions ranging from minor discomfort, such as skin depigmentation, to severe conditions such as pancreatitis, colitis, or lung and/or liver toxicity [57]. Further, persistent inflammation can pave the ground towards the development of an AD [58]. Taken together, an inflammation can potentially progress towards neoplasia or an AD, and this process can be facilitated by a dysregulation of the innate immune system [50,53].

### 4.1. Melanoma and Autoimmune Disease

The interaction between cancer and ADs is evident in melanoma patients which becomes obvious in the inverse relationship between melanoma and vitiligo. Briefly, the disorder is attributed to the production of autoantibodies against immunogenic, melanocytic-specific molecules (melanocytic-differentiation antigens, MDAs) that subsequently attack melanocytes resulting in white skin patches. In this context, antibodies against melanocytes provide natural protection against cancer and decreasing the risk of melanoma in vitiligo patients [59]. In contrast, a similar antibody-based condition called melanoma-associated hypopigmentation (MAH) or vitiligo-like depigmentation develops in some melanoma patients. Usually, this is considered a predictor of improved therapy outcomes [60], although some studies found that hypopigmentation can be associated with disease progression [61,62]. In the latter case, a DNp73/IGF1R/Slug signature in colorless lesions might aid to clinically distinguish patients with MAH-associated metastatic melanoma from those, where MAH is indeed a sign of regression [62]. Considering that malignant melanoma co-evolves with immune cell phenotypes [63], this dynamic interaction between autoimmunity-induced hypopigmentation and skin cancer may become a double-edged sword in the long-term. In particular, these tumors exhibit high intratumoral heterogeneity and plasticity [63]. Therefore, over the course of the disease, strict immune surveillance against MDA-expressing cells could serve as a microenvironmental cue that exerts evolutionary pressure for immune selection of cell variants with low-MDA expression that are poorly recognized by autoantibodies. These MDA-negative cells characteristically show enhanced invasive abilities. Thus, anti-MDA responses provoked by the melanoma tumor can in turn, promote clonal expansion of low-MDA-expressing cell variants with activated pro-metastatic programs that can migrate to distant sites, giving rise to secondary tumors [62].

Moreover, other autoimmune comorbidities positively correlate with metastatic melanoma [33,63]. A retrospective meta-analysis assessed that the prevalence of pre-existing AD in melanoma patients increased by 1.7-fold within a decade. Prevalence rates were higher in metastatic skin cancer patients compared to primary, non-metastatic melanoma patients or the general population, suggesting that a pre-existing AD could possibly favor cancer aggressiveness. The most common ADs in patients with metastatic melanoma were myositis, peripheral neuropathy, RA, psoriasis, autoimmune pancreatitis, type 1 diabetes mellitus, autoimmune aplastic anemia, relapsing polychondritis, Hashimoto encephalopathy, and IBD. One possible reason could be perturbations in common molecular or immunological pathways that lead to an increased susceptibility of AD patients to melanoma [33]. Another clinical study showed that only a small percentage of melanoma patients have preexisting AD. Interestingly, these patients had a significantly shorter median overall survival and disease-free survival after initial metastasis compared to patients with a primary tumor, and that the worse prognosis was independent of AD treatment-related side effects. Patients with antibody-mediated AD had a poorer prognosis than those with T cell-related AD [64]. In summary, these data reveal a potential connection between malignant melanoma and immune system dysregulation, implying that a pre-existing AD may be a predisposing factor for melanoma onset and progression. If that is indeed the case, further experimental investigations are required.

### 4.2. AD, Cancer, and Immunotherapy—An Underinvestigated Conncetion: Hurdles or Chances?

To effectively treat ADs, numerous clinically approved antibodies are at hand [65,66,67,68,69,70,71,72,73]. However, their use is often associated with side effects, including the development of cancer. Natalizumab has been reported as causative for the development of the JC-virus-mediated progressive multifocal leukoencephalopathy (PML) [74] and is also connected with a potential risk for the onset of melanoma [75]. Unique secondary ADs such as thyroid disorders, immune thrombocytopenia, and nephropathies have been observed for alemtuzumab [76,77], while papillary thyroid carcinomas and melanomas appeared to be the most common cancers [76].

A major breakthrough in the treatment of AD was the development of anti-TNF antibodies (e.g., infliximab, adalimumab). As a main regulatory cytokine, TNF plays a central role in inflammation and autoimmune diseases [78], and anti-TNF therapy dramatically improves clinical symptoms and quality of life in AD patients. However, since pro-TNF therapy was initially intended to treat cancer, concerns about adverse effects accompany the anti-TNF therapy. In the treatment of RA, anti-TNF therapy was found to increase the risk of cancer and severe infections [79], while anti-TNF therapy for Crohn’s disease has been reported to be correlated with a significantly higher risk of lymphoma [80]. Overall, the most frequent cancers related to this therapy are lymphoma and melanoma, and the most common infection is pneumonia [81,82,83]. Interestingly, patients with non-biologic drug-resistant ulcerative colitis and breast cancer can be well treated through TNF blockade without tumor progression [82]. Although anti-TNF therapies remain a cornerstone in the treatment of ADs, optimal management is needed to handle the serious associated issues, including the development and progression of cancer, underscoring the growing importance over controlling the immunoreaction.

Indeed, cancer and AD represent two different pathological conditions with opposite immunity patterns, but nevertheless, there is plenty of evidence suggesting a bidirectional association between both diseases [84]. This fact is even more crucial for ICI treated cancer patients with pre-existing ADs (PADs). ICIs enhance anti-tumor immunity via improved immune stimulation targeting blocked immune regulatory pathways [85]. However, activation of the immune response by ICIs is also associated with a wide range of irAEs. They occur most commonly in the skin, liver, thyroid, and gastrointestinal tract but can also affect most other organ systems. In particular, when ICI therapy is considered in patients with PAD, especially when the mechanism of action of the drug is closely related to the pathophysiology of PAD, it may lead to exacerbation of the autoimmune disease [86]. Furthermore, affected patients appear to be predisposed to a more severe course of irAEs having an underlying abnormal immune response to self-antigens. Consequently, cancer patients with autoimmune diseases have usually been excluded from clinical trials and informations are mostly received from case series/case reports on the course of specific PADs associated with ICI therapy, unfortunately limiting the available data [49,86,87,88,89,90,91]. Accordingly, the efficacy and safety of PD-1/programmed cell death ligand 1 (PD-L1) inhibitors in cancer patients with AD remain poorly understood [54].

Moreover, there are concerns about the use of immunosuppressants such as corticosteroids at the beginning of treatment with PD-1/PD-L1 inhibitors in AD patients, since they may impair the efficacy of the immunotherapy in these patients, exacerbate the existing disease, or alter the risk of new irAEs. In a recent report, Tison et al. (2019) studied the efficacy and safety of ICIs in a cohort of 112 patients that suffered from both, cancer and PAD [87]. They observed that 71% of the patients experienced immune toxicity, while almost one half suffered from a PAD flare or developed an irAE unrelated to the PAD (47% and 42%, respectively), and another 18% of patients experienced even both. Based on these conditions, the majority of patients with PAD and/or irAEs were treated with immunosuppressants. Eventually, the authors summarized that flares or irAEs frequently occur in patients under ICI treatment and that they are mostly manageable without discontinuation of the therapy. In a strong contrast, they also found that the occurrence of irAEs were associated with worse cancer outcomes and that application of immunosuppressants at the beginning of ICI treatment appeared to have a negative impact on the therapy progress. Another retrospective study was performed on patients with advanced melanoma and PAD who had received a combination of anti-CTLA-4 and anti-PD-1 therapy. Particularly in patients with inflammatory bowel disease, rheumatologic conditions, and patients on baseline immunosuppression showed a higher risk of flare of PADs. Moreover, patients treated with immunosuppressants had a reduced overall survival rate of 11 months compared to 31 months of untreated patients [92]. Similar results were described by van der Kooij et al. (2021) who investigated the response of anti-CTLA-4, anti-PD-1, or a combination therapy in advanced melanoma, highlighting that among patients with pre-existing IBD, severe colitis and toxicity more frequently occurred, thus requiring an early discontinuation of the treatment [93]. Considering these needs, drug repurposing could improve the development of immunotherapies and provide more rapid solutions.

## 5. Prospects of Drug Repurposing

In traditional drug discovery, new molecular entities (NMEs) are being identified and developed de novo for precision medicine, largely based on the “lock-and-key” specificity. It is assumed that newly developed molecules targeting a certain signaling pathway will highly selectively destroy the tumor, maximize efficacy, and minimize side-effects [94]. In practice, however, the results are often less satisfactory. Many innovative drugs with a promising profile in preclinical settings prove to be insufficiently effective and/or unsafe in clinical use. In developing new drugs from scratch, failure rates are discouragingly high and disproportionate to the time and costs involved [95,96]. In this sense, drug repurposing which refers to the discovery, validating, and marketing of already approved compounds for new indications outside the scope of the original medical use, promotes a paradigm shift in drug discovery and development [97]. In the current scenario, the efforts of drug development have been significantly reduced, with the risk of failure additionally lowered by the advancement of bio- or cheminformatics tools and the availability of vast biological and structural databases for drug repositioning.

### 5.1. Repurposed Drugs in the Context of Anti-Metastatic Treatment and Cancer Immunotherapy

A large number of preclinical studies have identified more than 200 conventional drugs with off-label anti-tumor effects and excellent reviews on this topic have been published recently [98,99,100,101]. Here, we summarize information about the most promising non-oncological repurposed drugs on track for clinical use that target receptors, signaling pathways, and proteins linking cancer hallmarks and microenvironment crosstalk.

#### 5.1.1. Niclosamide

Several research groups demonstrated that niclosamide, initially EMA- and FDA-approved for anti-helminthic treatment, has a strong potential for alternate use as anti-cancer drug. The substance interferes with tumor progression and metastases formation via S100A4 inhibition. Liu and colleagues showed that niclosamide combined with cisplatin exerts its anti-cancer effects in triple-negative and chemoresistant HER2-positive breast cancer by reversing EMT and inhibiting stemness and invasion [102]. Furthermore, the drug also blocks metastasis in hepatocellular carcinoma cell lines by downregulating Twist-mediated CD10 expression [103]. The molecular mechanisms underlying the effects of niclosamide in malignancies are divers and related to its interference with crucial cancer pathways such as PI3K/Akt, Wnt/beta-catenin, Jak/STAT, and NF-κB signaling, which have been identified as potential targets in different cancers [101]. Another preclinical study revealed that this agent lowers the growth of melanoma cell lines and induces mitochondrial apoptosis, which impairs cell migration and invasion, reduces expression of phosphorylated STAT3 at Tyr705, and inhibits matrix metalloproteinase-2 and -9 expression [104]. In terms of the suitability of repurposed compounds to improve cancer immunotherapy, niclosamide in combination with PD-1/PD-L1 antibody has a synergistic anti-tumor effect in non-small cell lung cancer models through decreasing PD-L1 expression and promoting cytotoxic T cell activity [105]. Specifically, niclosamide-dependent PD-L1 downregulation is related to blockade of STAT3 phosphorylation and its binding to the PD-L1 promoter. Although no clinical studies have been reported in the context of melanoma, a phase II trial has been previously conducted to investigate the safety and efficacy of orally applied niclosamide in patients with colorectal cancer metastases ([106], NCT02519582).

#### 5.1.2. Aspirin

Aspirin is a prototype non-steroidal anti-inflammatory drug to treat various pain and inflammatory disorders. The compound also suppresses blood clotting by inhibiting the physiological function of platelets and is administered to prevent heart attack and stroke. A new use of aspirin aims at manipulating anti-cancer immunity in the TME based on its efficiency as antiplatelet drug. According to a recent report, platelet activation represents a mechanism of immune evasion that mediates suppression of CD8^+^ T cell function within the TME [107]. Pharmacological treatment of platelet function with aspirin enhanced responsiveness to PD-1 blockade. Similar results have been obtained from breast cancer patients, in which aspirin therapy reduced tumor cell IL-8 secretion, and the metastatic phenotype [108]. Further, aspirin supports the elimination of tumor cell debris generated during cancer therapies by activating macrophages and blocking secretion of pro-inflammatory cytokines from the decaying cancer cells [109]. In addition to macrophages, aspirin also exerts an immunomodulatory function on other immune cells such as MDSCs and Tregs [101], overall proposing aspirin as an attractive agent for combination therapies. In fact, this could already be demonstrated in a clinical trial for the treatment of cervical and uterine cancer. The mixture of aspirin with vitamin D, cyclophosphamide, and lansoprazole forms an immunomodulatory cocktail that can be combined with pembrolizumab and radiotherapy ([110], NCT03192059). Moreover, a phase II clinical trial has been completed recently, investigating the combination of ipilimumab/pembrolizumab and aspirin in patients with metastatic melanoma (NCT03396952).

#### 5.1.3. Denosumab

Denosumab is an inhibitor of the receptor activator of nuclear factor kappa B (RANK) ligand (RANKL) and has been approved for therapy of skeletal-related events in patients with advanced conditions including solid tumors and multiple myeloma. Mainly responsible for bone homeostasis, the RANK/RANKL system regulates bone remodeling and osteoclast function [111]. Further insight revealed the impact of RANK/RANKL on tumor initiation, progression, and metastasis as well as its role at the interface between bone and the immune system. Specifically, RANK/RANKL signaling can influence immune functions such as dendritic cell survival, macrophage activation and T-cell activation and differentiation [112]. Based on the role of RANK/RANKL and its involvement in cancer biology, denosumab has been successfully repurposed for the treatment of a rare disease, the giant cell tumor of bone [112]. Currently, some phase II clinical trials evaluate combination of desmosumab and ICIs for the treatment of metastatic melanoma (NCT03161756: ipilimumab/nivolumab/denosumab; NCT03620019 pembrolizumab/nivolumab/denosumab).

#### 5.1.4. Metformin

Metformin, an oral antidiabetic drug commonly taken by patients with type 2 diabetes mellitus, was developed as cancer therapeutic and is currently in Phase II/Phase III clinical trials investigating its combination with immune checkpoint inhibitors in advanced melanomas (NCT03311308: pembrolizumab/metformin, NCT01638676: vemurafenib/metformin, NCT02143050: dabrafenib/trametinib/metforin) [100,113]. The anti-cancer activity of metformin is mediated particularly through direct inhibition of the AMPK/mTOR pathway and indirectly influenced by its glucose-lowering properties and anti-inflammatory effects [114]. Metformin has been reported to enhance immunotherapy via multiple effects on the tumor immune microenvironment, including protection of CD8^+^ T cells from apoptosis, depletion of PD-L1, and reduction of intratumoral hypoxia, together preserving anti-tumor immune cell functionality and reversing an immunosuppressive TME [100]. A previous study found that the efficacy of PD-1 blockade is potentiated by metformin-induced reduction of tumor hypoxia [115]. The PD-L1 expression level in tumors is considered a critical factor of clinical response to ICI therapy [116]. Expression of the PD-L1 gene is influenced by several factors such as TP53 and retinoblastoma (Rb) protein [117]. Cha et al. (2018) showed that in breast cancer metformin promotes anti-tumor immunity by endoplasmic reticulum-associated degradation of PD-L1 [118]. In endometrial cancer, metformin blocks PD-L1 expression in an AMPK-dependent manner [119]. These studies suggest that the drug could serve as an add-on therapy to enhance the anti-tumor effect of PD-1/PD-L1. Like metformin, phenformin belongs to the same biguanide class and showed even better anticancer activity compared to metformin, especially in BRAF-inhibitor resistant melanoma. In vivo studies showed that the combination of the BRAF inhibitor vemurafenib with phenformin activated AMPK pathway and induced apoptosis.

Other repurposed drugs such as rapamycin analogs, galloflavin, dichloroacetate, azaserine, and the MCT inhibitor lenalidomide that modulate various metabolic pathways in cancer, small molecules that cause reversal of T cell exhaustion, and antiestrogens have emerged as viable options for reprogramming the TME and to overcome immunotherapy resistance [100]. Further examples of drugs with off-target profile that proved useful for metastases intervention include the glutamic acid derivative thalidomide, originally used as sedative and antiemetic, which has immunomodulatory, anti-inflammatory, and antiangiogenic properties via inhibiting VEGF, bFGF, and TNF-α [120]. Among the repurposed agents to target metastatic dissemination, berberine (BBR) should also be mentioned. BBR is a bioactive compound traditionally used in Chinese and Ayurvedic medicine that prevents tumor cell invasion and metastasis by downregulating EMT and metastasis-related protein expression [121]. For example, BBR inhibits MMP-2 and MMP-9 expression by downregulating TGF- β1 and the COX-2/PGE2–JAK2/STAT3 axis in breast cancer [122,123], and epithelial-mesenchymal transition in melanoma by inhibition of the RARα/β-mediated PI3K/AKT signaling pathway [124]. Suppression of melanoma skin cancer cell migration and invasion after BBR treatment is associated with reduction of SOS-1, p-AKT, MMP-1, NF-κB, Ras, p-FAK, and MMP-13 gene expression and an increase in the levels of PI3K and PKC [125]. In addition, BBR suppresses IL-6-induced STAT3 activation in nasopharyngeal carcinoma cells and by tumor-associated fibroblasts [126].

### 5.2. Perspectives of Drug Repurposing at the Interface of Cancer, AD and Immunotherapy

The treatment of inflammatory diseases essentially consists of pharmacologically counteracting the deregulated immune system. For many years, this goal was achieved through application of glucocorticoids and conventional immunosuppressants such as methotrexate or cyclophosphamide. Due to their pleiotropic activity, these drugs need to be balanced against their potential toxicity for an effective use in patients [127]. With the considerable progress in understanding the pathogenesis of inflammatory diseases, targeted therapies have become available and drug repurposing has taken a crucial place in this development. Initially, pharmacological agents were repurposed based on unexpected clinical observations and relied on putative disease similarity. Many of the current standard therapies to treat RA inflammatory conditions have been repurposed from another rheumatoid or unrelated diseases. These have been first tried with particular success for RA following ankylosing spondylitis and psoriatic arthritis. Contrary, drug repurposing has been less successful for SLE and primary Sjörgen syndrome, which was related to disease heterogeneity, multiple organ involvement, and problematic results variables in clinical trials. Nevertheless, repurposing remains an important method to develop new treatments [128].

High-throughput approaches together with genome-wide association studies integrating disease-associated variants including diverse genomic and biological data allowed deeper insight into the complexities of disease pathogenesis and elevating drug discovery. In this regard, Okada et al. (2014) performed such a meta-analysis of more than 100,000 patients identifying 42 novel risk loci, 98 genes and related pathways underlying RA. Importantly, they demonstrated that these genes were targets of approved RA therapies, and further suggested repurposing of approved drugs for other indications, including breast cancer, lymphoma, or hepatocellular carcinoma [129].

Multiple sclerosis (MS) is an autoimmune disease possibly caused by Epstein-Barr virus. It occurs as a combination of genetic variations, environmental factors, and/or the viral infection and leads via deregulation of the immune system to severe demyelination, neurodegeneration, and the formation of lesions in the white and gray matter of the spinal cord and brain [130,131,132]. Due to the disease’s complexity, classical drug development has so far failed to identify curative drugs, thus causing a strong limitation in therapeutic options. Here, repurposing emerged as a useful alternative for rapidly approving potential agents already clinically approved for other indications. Recently, Amadio et al. (2022) established SAveRUNNER, a network-medicine-based algorithm for drug repurposing and analyzed the human interactome focusing on the interplay between MS-associated genes and drug targets. As a result, these authors were able to find new histamine drug-disease associations and could predict off-label use of the histaminergic drugs amodiaquine, rupatadine, and diphenhydramine as novel potential MS therapeutics [133].

Similarly, in-depth analyses of RNA-seq data from SLE patients were used to establish molecular endotypes, which represent modules of commonly regulated genes. This molecular taxonomy-based pipeline of Garantziotis and colleagues (2022) highlighted five lupus endotypes, each characterized by a unique gene module enrichment pattern, which in consequence also allowed re-stratification of SLE patients through a hierarchical clustering [134]. Based on these insights they performed drug repurposing analysis to identify perturbagens that counteract group-specific SLE signatures as well as for their ability to reverse the gene expression signatures in each molecular endotype, finally identifying four potential subgroup-specific approved compounds, namely bortezomib (neutrophilic cluster), azathioprine/ixazomib (B cell cluster), and fostamatinib (metabolic cluster). Although experimental validation is still needed, it illustrates the benefits of systemic high-throughput computational methods to optimize putative therapeutic choices for personalized immuno-oncology [134].

**Figure 1:**
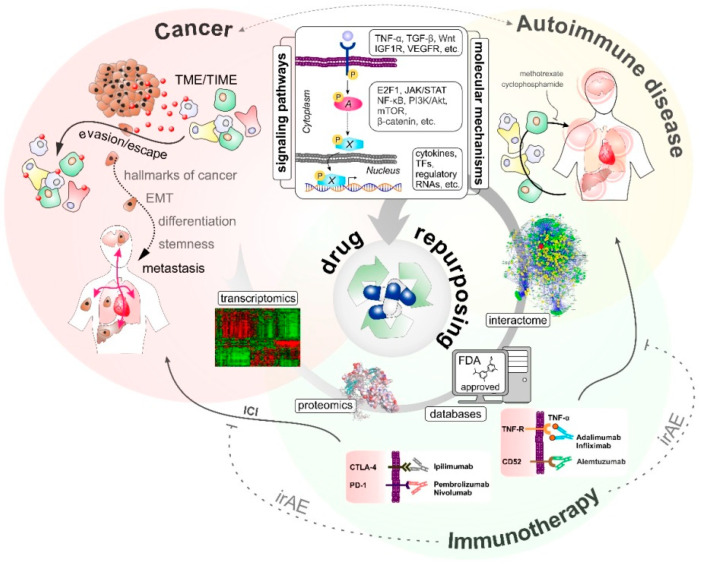
Drug repurposing at the interface of cancer, autoimmune diseases, and immunotherapy.

In summary, the profound data availability for ADs led also to the discovery that many autoimmune diseases share similar inflammatory pathways with common molecular mechanisms [135,136,137]. Above all, this overlap is reflected in the broad efficacy of the inhibitor TNF-α used for the treatment of RA, psoriasis, or IBD [97]. In this context, the JAK-STAT pathway could be identified as a mutual major target of AD therapies. This pathway is of particular interest for treating RA and SLE, since inhibition of JAK1 blocks both, IL-6 and type I IFN signaling, two cytokines well known for their role in the pathophysiology of both diseases [127]. Thus, knowledge of molecular and mechanistic overlaps between different ADs and other malignancies offers the possibility of drug repurposing for targeted therapies across these diseases. Strategies for drug repurposing at the interface of melanoma immunotherapy and ADs are summarized in Figure 1.

## 6. Computational Approaches for Drug-Repurposing

In recent years, drug repurposing research has played an important role and benefited greatly from the systematic adoption of computational strategies covering a wide range of unique methods and approaches. To gain insights into the disease state and the underlying cancer signaling pathways, molecular modeling of therapeutic protein targets lets in for the information of the structural biological characteristic as well as “virtual high-throughput screenings” to identify innovative drug candidates. In particular, molecular docking [138] and omics-based [139] strategies are useful tools in drug repurposing research. The basic principle behind repositioning is to evaluate drugs with similar chemical structures and biological activities to determine whether they may have similar clinical indications. The similarity of protein structures at local ligand binding sites has been exploited to discover pills to target illnesses beyond their authentic warning signs. inside the public domain, many unique databases, along with Drug Repurposing Hub [140], Drug Goal Commons [141], and Open Targets [142], have been recognized to integrate various computational methods and allow searches for repurposed drugs using complete facts. The maximum latest databases repoDB [143], ZINC15 [144], and repurposeDB [145] covered the data of the clinical effects of drug repurposing. Schneider and his team are investigating a comprehensive visual analysis tool called ClinOmicsTrailbc that helps analyze clinical biomarkers, genomics/epigenomics, and transcriptomics datasets to identify targeted drugs, and drug candidates for repurposing, and to provide and facilitate a holistic evaluation of the use of immunotherapy in the therapy of breast cancer [146].

In drug repurposing, mainly two steps are required. The first step involves virtual screening of clinically approved or marketed drugs that are effective against a particular therapeutic target. Once shortlisted drugs have been identified, they are investigated using in vitro and in vivo methods in specific pathophysiological pathways of the disease in question. In the second phase of repurposing, clinical studies are conducted for the respective indication.

### 6.1. Traditional Drug Discovery versus Drug Repurposing

In the past, drug discovery had involved de novo identification and development of NMEs. This process includes the five phases: discovery and preclinical assessment, safety review, clinical research, FDA review, and FDA post-marketing safety monitoring. In contrast, drug repositioning has only four phases, namely compound identification, compound acquisition, development, and FDA post-marketing safety surveillance. In this current scenario, the time and cost of drug development have been significantly reduced, with the risk of failure reduced due to the advancement of bioinformatics/cheminformatics tools, the availability of huge biological and structural databases in drug repositioning and artificial intelligence technology leads to enormous acceleration of the drug repurposing process. Approaches and applications of AI and machine learning have been recently published in some excellent reviews [147,148,149,150,151].

### 6.2. Strategies of Drug Repurposing

With the help of public databases of drug/chemical libraries, computational biology and bioinformatics/cheminformatics tools were used as a source for virtual screening. Using this approach, molecular interactions between drug molecule and protein target that function as potentially bioactive molecules were identified [152]. Based on the information available for the pharmacological, toxicological, and biological activity in the public domain, drug repurposing methodologies were divided into two parts, namely drug-oriented and the target-oriented strategy, respectively. The methodology of drug-oriented testing includes the structural features of drug molecules, biological activities, adverse effects, and toxicities, and these characteristics are used to identify molecules with biological effects based on cell/animal studies. These approaches are based on the traditional principles of pharmacology and drug discovery, which consider the biological efficacy of drug molecules without really knowing the biological targets. Alternatively, target-oriented strategies rely on in-silico or virtual high-throughput screening (vHTS) of drugs/compounds from various drug libraries/compound databases. In the latter one, most biological targets directly represent the disease pathways/mechanisms [153].

#### 6.2.1. Drug-Based Strategies

**Figure 2:**
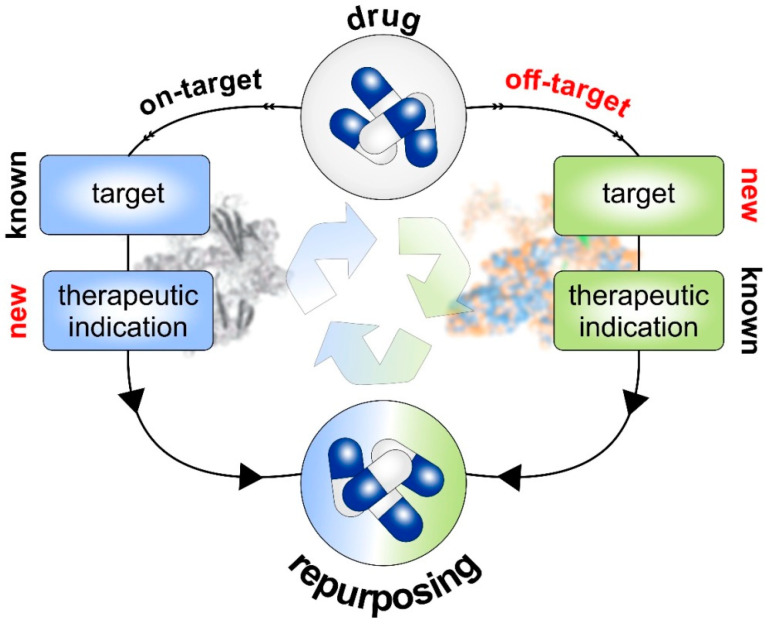
On-target and off-target strategies of drug repurposing. The on-target pipeline only considers drugs with known target(s). Once the target is identified and validated in the patho-physiology of new disease therapeutic indication, the drug may also be repurposed for that. In case of off-target strategies, drugs are screened against already prioritized therapeutic targets of disease under investigation.

The drug-based strategies mainly focus on drug-related data such as chemical, molecular, biomedical, pharmaceutical, and genomic information as the basis for predicting therapeutic potential. Drug repurposing can be achieved by the on-target strategy or the off-target strategy (Figure 2). This process is based on the substantial drug-related data accessible or significant motivation for investigating how pharmacological characteristics can contribute to drug repositioning. On-target repositioning involves applying the known pharmacological mechanism of a drug molecule to a biological target for a new therapeutic indication. A representative example is minoxidil. The on-target profile of this drug consists of acting on the same target and producing two different therapeutic effects, thus, repositioning minoxidil from an antihypertensive vasodilator to an anti-hair loss drug. By lowering blood pressure, minoxidil dilates blood vessels and opens potassium channels. Thereby it enhances blood flow and, consequently, more oxygen and nutrients reach the hair follicles eventually favoring the splendor of hair especially to the delight of the human male.

In off-target profiling, the pharmacological mechanism of a drug is unknown, so drugs and candidates act on new targets that fall outside the original scope of the drug. As described above, aspirin is a good example for a drug with an off-target profile. Several publicly available databases offer extensive information on lead molecules and their interactions which can be used and integrated in different drug repurposing strategies (Table 1).

#### 6.2.2. Target-Based Strategies

**Figure 3:**
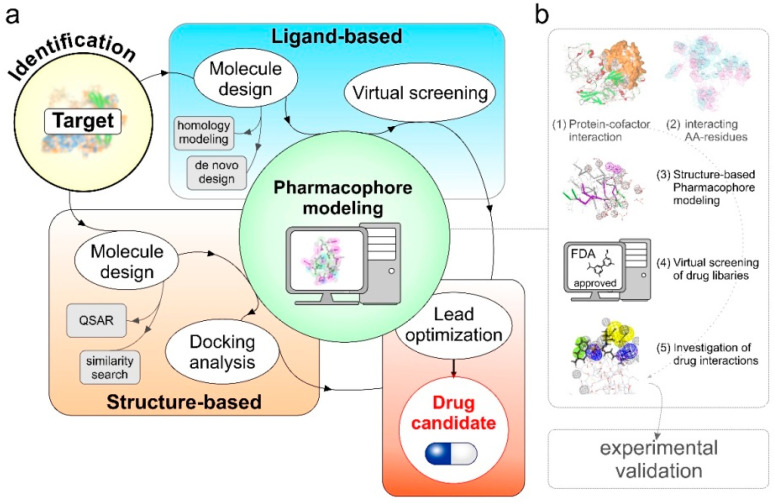
Overview of drug candidate screening from target identification including the structure-based pharmacophore modeling workflow towards therapy personalization. (**a**) In the target identification two processes were involved, namely structure-based and ligand-based. Different methods were used to design the molecules, such as homology modeling, de novo design, and QSAR. After completion of the modeling part, the models were subjected to pharmacophore feature identification of the molecules. Following docking and virtual screening, models were optimized to extract the drug candidate. (**b**) (1) Protein-cofactor interaction analysis was performed to identify to best binding pose using PP docking methods. (2) In the best binding pose, amino acid residues involved in the direct interaction between both interacting molecules were identified. (3) From selected amino acid residues, pharmacophore features were identified to design the structure-based pharmacophore model. Two hydrophobic groups are shown as violet spheres; two hydrogen bond donors (green arrows); two hydrogen bond acceptors (pink arrows); and twelve excluded volumes (gray spheres) are shown. The peptide backbone is highlighted as a thick stick model. (4) In the next step, FDA approved drug library were screened for the small molecule inhibitors that bind with the predicted pharmacophore and interfere its interactions with the cofactor. (5) Identification of suitable compounds among the top screened drug mapped to the 3D pharmacophore query space shown as meshed sphere. Finally, experimental validation is needed to verify and test the functionality of the predicted drug.

In contrast to drug-based repurposing approaches that start with chemical compounds, target-based repurposing settings use the prioritized target or biomarker of the disease under investigation. The target is used to screen drug compound libraries using high-throughput and/or high-content screening (HTS/HCS) using computational methods such as ligand-based screening or docking [154] (Figure 3a). Compared to the other methods that do not use biological targets or pharmacological information for screening, target-oriented repurposing directly links to the protein responsible for the disease condition and therefore, greatly improves the likelihood of drug discovery.

## 7. Pharmacophore Modeling towards Therapy Personalization in Melanoma

As explained earlier, E2F1 and its interacting coregulators are important therapeutic targets to combat melanoma metastasis. Since disruption of the malignant associations might restore E2F1’s ‘bright-side’ towards an anti-metastatic outcome, characterization of the E2F1 coregulome emerges as a need in terms of developing novel therapies. A high-throughput Co-IP-mass spectrometry approach to screen for E2F1:coregulator interactions in metastatic cells of various cancer types was employed. We found that the metastasis-associated protein 1 (MTA1) which turned out to be a direct E2F1 target gene, forms a complex that synergistically potentiates hyaluronan synthase 2 (HAS2) expression, leading to extracellularly increased HA production and enhanced cell migratory and invasive capacity [16]. Dissociation of this pro-metastatic circuit by targeting E2F1:MTA1 assembly reduced tumor-associated macrophage infiltration in the TME. Using these data, structure-based pharmacophore modeling of FDA-approved marketed drugs from a virtual library revealed that the small molecule compound argatroban is a potent inhibitor of the E2F1:MTA1 complex. As demonstrated by Goody et al. (2019), treatment of E2F1/MTA1-positive, highly aggressive, circulating melanoma cells with argatroban resulted in prevention of metastases [16]. Even more impressive, in a clinically relevant orthotopic mouse metastasis pancreatic tumor model, argatroban treatment led to cancer relapses. This occurred via perturbation of the E2F1:MTA1/HAS2 regulatory axis [16]. Argatroban is a reliable and predictable anticoagulant that binds reversibly and selectively to the thrombin active site and inhibits thrombin-catalyzed or -induced reactions, including fibrin formation, activation of coagulation factors V, VIII, and XIII, activation of protein C, and aggregation of platelets. The compound is currently prescribed against heparin-induced thrombocytopenia and for use in patients undergoing percutaneous coronary intervention [155]. However, based on its newly identified function and considerable in vivo effectiveness, repositioning of argatroban offers a genuine therapeutic solution to combat metastatic cancers that could be brought to bedside quite rapidly. Moreover, drug safety data from Phase I and II clinical trials of argatroban are already in place [156].

### 7.1. Pharmacophore Modeling

Once a protein and its tumor metastasis driving coregulator complex is known, small molecule inhibitors can be screened using pharmacophore modeling approaches (Figure 3a). In pharmacophore modeling, the three-dimensional arrangement of essential chemical features, for example, hydrogen bond-donor, -acceptor, positive/negative ionizable atoms, hydrophobic and aromatic groups, are analyzed from the active site and/or binding interface of protein–coregulator complex that mediate biological activities [157]. Depending on the target-drug information available in public databases, pharmacophore modeling approaches can be divided into three main categories:

1. Predictive-pharmacophore: when the list of drug molecules benefiting a clinical phenotype is known but the related target information is missing. These methods learn from the chemical features present in active and inactive compounds and quickly screen a virtual library of similar compounds for drug repositioning [158,159].
2. Receptor–ligand pharmacophore: when the information about the drug and target is known, these methods generate pharmacophore models using the features from receptor-ligand interactions [160].
3. Structure-based pharmacophore: these methods are suitable for the cases where the information about the potential therapeutic targets, protein–protein complexes, protein-cofactors driving this phenotype in known. The main goal is to find potential inhibitors that may fit to the active site or interfere in complex formation to benefit the clinical phenotype [161].

### 7.2. Argatroban as a Case Study

We used methods ranging from protein structure modeling, protein docking, and molecular dynamics simulation to prioritize amino acid residues participating in the formation of the binding interface which were in the line of experimental validations (Figure 3b). Using prioritized amino acid residues, we then developed a structure-based pharmacophore model to screen an FDA approved library of drugs and nutraceuticals of ~2900 compounds. We identified 16 compounds with the potential to fit our pharmacophore model. For the selected compounds, we then performed molecular docking and detailed molecular dynamics simulation studies and found argatroban that interacts with MTA1 and interferes with complex formation with E2F1. Our findings were successfully validated using in vitro and later in vivo mouse models. Using structure-based pharmacophore modeling, we were not only successful in repurposing argatroban as an anti-metastasis drug but also able to provide the underlying mechanisms of its action. Importantly, with the discovery of a large number of E2F1:coregulator complexes that drive metastasis, patient-specific pharmacogenomics profiling of cofactors combined with small molecule inhibitors identified using pharmacophore modeling approaches may improve personalized cancer therapy.

#### 7.2.1. Structure Modeling and Quality Assessment of E2F1 and MTA1 Proteins [9,11]

We used threading-based homology modeling approach to design the 3D structures of E2F1 and metastasis associated protein (MTA1). There are several web-based and offline software tools available for the prediction of 3D structure of therapeutic targets. Recently, alpha fold predicted the 3D structure of protein, using the primary amino acid sequence and aligned sequences of homologues as inputs. This tool is based on the AI program developed by Alphabet’s/Google’s DeepMind which performs predictions of protein structures. In our previous work, we used iterative threading assembly refinement (I-TASSER) server that uses composite modeling approaches combining various techniques such as threading, ab initio modeling, and atomic-level structure refinement, and provided a list of potential templates to be used in the modeling procedure [162,163,164]. Before analyzing the binding interface between TF-cofactor complex for the drug repurposing, the best models need to the optimized for loops and side chains. Tools such as Looper and ChiRotar can be used for this purpose. While Looper searches low CHARMm energy loop structures using a minimal representative set of the possible backbone conformers [165], ChiRotor is based on systematic searching of side-chain conformation and CHARMm energy minimization [166].

#### 7.2.2. In Silico PPI Docking Studies Suggest MTA1-E2F1 Physical Interaction

We performed PPI docking studies to find potential interaction poses between E2F1 and MTA1 using ZDOCK and RDOCK protocol [167,168] available in Biovia Discovery Studio suit. We analyzed the top 100 interaction poses and prioritized interacting amino acid residues between these two proteins. Selection of the amino acid residues are critical for the structure-based pharmacophore modeling to screen drug libraries for drug repurposing. Here the information from experimental methods such as site-directed mutagenesis, information from protein functional domain can also be important. In the case of E2F1 and MTA1, we observed that most of the residues from E2F1 targeted by MTA1 are from DNA binding and transactivation domains that play a crucial role for the transcription factor activities of E2F1 (Figure 3b, steps 1 and 2).

#### 7.2.3. Screening of Non-Peptidic Small Molecule Inhibitors Disrupting E2F1-MTA1 Interaction in Metastatic Tumor Cells Using Pharmacophore Modeling

To disrupt the interaction between E2F1 and MTA1, we selected key amino acid residues of MTA1 and prepared pharmacophore models using ‘create pharmacophores’ protocol available in Biovia Discovery Studio software suit considering various features including H-bond donors, H-bond acceptors, hydrophobic groups and excluded volumes. The 3D pharmacophore model designed was further used as an input query for virtual screening of ZINC drug database (Zdd) which is a collection of 2924 compounds in the categories of drugs approved for use in human and neutraceuticals commercially available as pure compound (ZINC subset ID: 96). We used ‘screen library’ protocol of Biovia Discovery Studio with the flexible fitting methods to find the best confirmations of filtered ligands in the pharmacophore 3D query space. Out of 2924 compounds, we found 16 potential drugs that can bind to the selected pharmacophore model. Among them, argatroban showed high efficacy to interact with MTA1 amino acid residues involved in the interaction with E2F1. Inhibition of E2F1-MTA1 complex formation using small molecule inhibitors through drug repositioning, identified an approved drug that potentially reduces metastasis (Figure 3b, steps 3–5).

## 8. Conclusions

Metastatic propensity is typical for melanoma and depends on molecular alterations in the cancer cells themselves and the immediate tumor immune microenvironment. This most aggressive form of skin cancer is therefore an excellent model system to develop targeted molecular pharmaceuticals and immunotherapies. Recent breakthroughs with checkpoint inhibitors have significantly increased the survival of patients with metastatic melanoma. Still, patients affected die from skin cancer and this underscores the need for new therapeutic strategies, particularly for those with preexisting AD or those who develop irAEs. Numerous research groups are searching for novel therapeutic vulnerabilities underlying metastasis in conjunction with the TME. Good progress has been made in the last decade primarily through the analysis of high-throughput data using bioinformatics and systems biology approaches. In this way, signaling network maps and protein interaction models of cancer cells have been generated that can be used for structure-based pharmacophore approaches and drug repurposing. Finding new therapeutic uses for existing drugs offers potential benefits for safe, affordable new treatments for cancer patients with high unmet medical need, easier access to clinical trials, and faster application and approval. However, due to a lack of economic incentives, pharmaceutical companies are rarely interested in conducting clinical trials of approved drugs to confirm promising preclinical results, especially once their basic patent or regulatory protection has expired. Consequently, funding of drug repurposing research and clinical validation must be put on a broader footing for instance by alternative academic or non-profit institutions to the benefit of patients in the future.

## Figures and Tables

**Table 1:** Summary of available drug/molecule databases applicable for different drug repurposing strategies.

Database	Description	Website
**Drug library databases**		
PubChem	Larger molecules such as nucleotides, polysaccharides, lipids, peptides, and chemically altered macromolecules are also present in the National Institutes of Health’s (NIH) open chemistry database alongside smaller molecules.	http://pubchem.ncbi.nlm.nih.gov
Drugbank	Contains information on drugs and drug targets. Database is a comprehensive, freely accessible, online. Created and maintained by the University of Alberta and The Metabolomics Innovation Centre.	http://www.drugbank.ca/
Chemspider	Chemical structures are included in databases, giving users quick text and structure search access to more than 100 million structures from countless data sources.	http://www.chemspider.com
ChemDB	Databases have all the commercially available important as well as small molecules which play important role for building useful blocks for drug discovery.	http://www.chemdb.com
ZINC	ZINC database contains over 230 million compounds for virtual screening.	https://zinc.docking.org
COCONUT	The collection of open natural products (COCONUT) database provides over 400 thousand natural compounds for virtual screening and drug repurposing.	https://coconut.naturalproducts.net
STITCH	STITCH databases provide known and predicted interactions between chemicals and proteins which can be used for drug repurposing.	http://stitch.embl.de
**Target 3D structure databases**		
RCSB Protein Data Bank (PDB)	Information of three-dimensional structural of large biological molecules, such as proteins and nucleic acids.	http://www.rcsb.org
OCA	Rapidly search through the contents of the entire PDB Archive.	http://oca.weizmann.ac.il/oca-bin/ocamain
Proteopedia	Structural and functional knowledge about biomacromolecules, their assemblies and interactions with small molecules.	http://proteopedia.org
**Drug-target databases**		
Drugbank	Data related to drug interactions, pharmacology, chemical structures, targets, metabolism and identify repurposing opportunities, or build predictive machine learning models.	http://www.drugbank.ca/
Pharmacogenetics Knowledge Base (PharmGKB)	Information on pharmacogenomics that is used to collect, organize, evaluate, and scientific information about how human genetic variation influences drug response.	http://www.pharmgkb.org/
Therapeutic Target Database (TTD)	TTD contains the information on the targeted disease, pathways, and known and under-researched therapeutic protein and nucleic acid targets.	https://bidd.group/group/cjttd/
Drug Target Commons	Improved consensus and utilization of drug–target interactions thanks to a crowdsourcing platform.	https://drugtargetcommons.fimm.fi/
Open Targets	Integrates public domain data to enable target identification and prioritization.	https://www.opentargets.org/
**Protein interaction databases**		
Human Protein Atlas	Central platform for all human proteins in cells, tissues, and organs using combination of various omics technologies, including antibody-based imaging, mass spectrometry-based proteomics, transcriptomics, and systems biology.	https://www.proteinatlas.org/
Biological General Repository for Interaction (BIOGRID)	Theme of the database is to curate on specific biological processes with disease relevance and curated for biological interactions.	http://thebiogrid.org/
Database of Interacting Proteins (DIP)	DIP include experimentally determined interactions be-tween proteins and collected a single, unified set of protein–protein interactions by combining data from many sources.	http://dip.doe-mbi.ucla.edu/dip/Main.cgi
STRING	Biological database and web resource of known and predicted protein–protein interactions.	http://string-db.org/
**Pathway databases**		
NCI Pathway Interaction Database (NCI-PID)	Relevant information related to pathway interaction of human cellular signaling and contained the molecular interactions and processes that occur in cells, with a special emphasis on actions that may be important for the study and therapy of cancer.	https://www.ndexbio.org
Kyoto Encyclopedia of Genes and Genomes (KEGG)	Collection of genomes, biological pathways, diseases, drugs, and chemical substances.	http://www.genome.jp/kegg/
PathwayCommons	Collection of the different biological pathways which includes proteins, DNA, RNA, and tiny molecules are involved in biochemical reactions, the building of bio-molecular complexes, transport and catalytic events, and physical interactions.	http://www.pathwaycommons.org/about/
REACTOME	Peer-reviewed pathway database of disease, signaling cascades, metabolic networks.	https://reactome.org
**Clinical trial information databases**		
Clinicaltrial.gov	The database includes international clinical trials that have been financed by both governmental and commercial sources.	http://clinicaltrials.gov
SIDER	Medicines that are marketed and their documented negative drug effects.	http://sideeffects.embl.de/
Drug Repurposing Hub	Collection of curated and annotated FDA-approved medications and pharmaceuticals used in clinical trials, and preclinical studies.	https://clue.io/repurposing
repoDB	This database contains successful and failed drug repositioning studies.	https://unmtid-shinyapps.net/shiny/repodb/
**FDA label information**		
FDALabel (US FDA)	Over 140,000 human pharmaceutical, biological, over-the-counter (OTC), and animal medicine labelling documents can be searched in a variety of ways using this application.	https://nctr-crs.fda.gov/fdalabel/ui/search
DailyMed (US FDA)	Contains 143,950 labeling submitted to the Food and Drug Administration (FDA) by companies.	http://dailymed.nlm.nih.gov/dailymed/about.cfm
**Omics data (Target/Drug)**		
NCBI-GEO	Store the gene expression profiling and RNA methylation profiling managed by the National Center for Biotechnology Information.	http://www.ncbi.nlm.nih.gov/geo/
Sequence Read Archive (SRA)	Repository of high throughput sequencing data.	http://www.ncbi.nlm.nih.gov/Traces/sra/
ArrayExpress	Contained functional genomics data which extracted from high-throughput functional genomics experiments.	http://www.ebi.ac.uk/arrayexpress/
Cancer Cell Line Encyclopedia (CCLE)	Detailed genetic and chemical characterization of over 1100 cancer cell lines.	http://www.broadinstitute.org/ccle/home

All websites were accessed on 20 December 2022.

## Data Availability

Not applicable.
